# Perceived Needs of The Family Caregivers of People with Dementia in a Mediterranean Setting: A Qualitative Study

**DOI:** 10.3390/ijerph16060993

**Published:** 2019-03-19

**Authors:** Sara Moreno-Cámara, Pedro Ángel Palomino-Moral, Lourdes Moral-Fernández, Antonio Frías-Osuna, Laura Parra-Anguita, Rafael del-Pino-Casado

**Affiliations:** 1 Department of Nursing, School of Health Sciences, University of Jaén, 23071 Jaén, Spain; smcamara@ujaen.es (S.M.-C.); afrias@ujaen.es (A.F.-O.); lparra@ujaen.es (L.P.-A.); rdelpino@ujaen.es (R.d.-P.-C.); 2 University Hospital “San Juan de la Cruz”, Úbeda, 23400 Jaén, Spain; lmf00006@red.ujaen.es

**Keywords:** caregivers, perceived needs, dementia, Alzheimer’s disease, nursing, qualitative research

## Abstract

The purpose of this study was to identify, classify and analyze the perceived needs of caregivers of elderly people with dementia during the care process. A descriptive phenomenological qualitative study using seven focus groups was conducted in different primary health care centers in the province of Jaén (Spain) between July 2012 and February 2013. Eighty-two family caregivers who were caring for people with dementia in different stages of the disease were selected by purposeful maximum variation sampling. Data were analyzed and organized thematically, considering the semantic and pragmatic content and field notes. Two main categories of the perceived needs of caregivers were identified. The first was related to the management of caring for a relative with dementia, and the second was related to the management of the caregivers’ own care. Our findings support the provision of comprehensive interventions for the improvement of caregivers’ emotional health that encompass more than one care need. This is where psycho-educational interventions aimed at managing the various aspects of dementia and self-care in caregivers can be accommodated. In addition, proactive interventions to develop important skills to care for a relative with dementia, which are not perceived as needs by the caregivers, are needed. These include skills in family negotiation, planning and searching for resources outside the family.

## 1. Introduction

The phenomenon of longevity is an unprecedented achievement in human history but, in turn, it is a major challenge. Providing satisfactory responses to the growing needs arising from the disability and dependency that accompany the ageing process is a great challenge for any modern developed society. This is especially so for societies with democratic and financially stable political organizations, where health care is considered a fundamental human right [1].

In general terms, humanity has gained not only in years but also in quality of life, but it is indisputable that diseases and, therefore, dependency and disability (and therefore care requirements) are more frequent in the elderly [2]. In this demographic and epidemiological scenario, chronic and degenerative diseases occupy a central place due to their incidence, prevalence and repercussions in terms of quality of life for the affected persons, as well as the consumption of resources and demands for care and attention for families and family caregivers [3].

Alzheimer’s disease, the most common cause of dementia, is also the leading cause of disability in older adults and the major contributor to dependency, financial overload and psychological stress in family caregivers [4,5,6].

Dementia is a chronic and complex disease that encompasses all conditions characterized by cognitive and functional impairment [7]. The management of this disease is a major challenge for the social and health policies of developed countries due to its incidence. In 2015, there were 46.8 million people with dementia in the world, and this figure is expected to almost triple by 2050, and high costs are involved [8], as well additional costs for the families who must assume care [9].

Family care, defined as unpaid support provided by family members or other people in the immediate environment of the caregiver, remains the mainstay of care for dependent older people in industrialized countries [10]. In fact, it is estimated that approximately 83% of the care received by dependent elderly people in Mediterranean settings takes place in the family context [10], validating the “Mediterranean or family-based model of care” [11] typical of southern European and Hispanic countries [12]. This model is characterized by a high family involvement in care and low participation by formal services [13].

Taking care of an elderly dependent person can affect the family caregiver’s health [14]. The high demands of such care are related to higher levels of subjective overload [15], anxiety [16] and depression [17,18]. However, caring for a family member with dementia can cause more physical and emotional problems than in other care situations, due to the high and changing demand for care from people with dementia [14,19,20].

Caring for a family member with dementia has been conceptualized as a dynamic and changing process [21]. Different studies have shown that cognitive behavioural problems and characteristics of dementia are the most consistent predictors of the negative consequences of care [17,22], which are related to the changing nature of the care needs of the affected person [23,24]. The scientific literature shows that support resources and interventions for family caregivers of elderly people with dementia do not seem to respond to their needs, and so stress accumulates, leading to burnout [7,25].

Knowing the needs perceived by family caregivers of people with dementia is essential for the proper care of such caregivers [7]. The latest specific review of the needs of these caregivers [26] highlights the broad, multidimensional and interrelated nature of the needs perceived by family caregivers of elderly people with dementia. The contributions of this review are based on research from countries whose caregiving model differs from the Mediterranean model due to the predominance of formal versus family support [27].

Several studies [11,28,29] have demonstrated the existence of a Mediterranean model of informal care characterized by (a) positive family attitudes toward the care of older relatives and, therefore, greater levels of family involvement in care, and (b) lower levels of formal caretaking (also in terms of coverage and services). Thus, in the Mediterranean context, the family assumes the care of the relative as an obligation and responsibility, which means that initially no help is sought from professionals [13,30,31]. Few existing studies have addressed this aspect specifically [32] and the needs of Mediterranean caregivers in general. In addition, most research on the needs of caregivers relies on quantitative methodology and on the perspective of professionals [33,34].

Therefore, more research is needed to encompass the experience of family care for elderly people with dementia to better understand the needs perceived by caregivers in Mediterranean settings. This information could guide the development of programs and interventions to ensure that the needs of caregivers are met.

The aim of the study was to identify, classify and analyze the perceived needs of family caregivers of elderly people with dementia during the care process in a Mediterranean setting.

## 2. Materials and Methods

### 2.1. Design

A descriptive phenomenological qualitative study was undertaken [35]. This allowed us to gain an in-depth understanding of the needs experienced by family caregivers of people with dementia during the care process. This method involved several stages of identifying, analyzing and reporting patterns of themes within data.

### 2.2. Participants

Eighty-two family caregivers of elderly people with Alzheimer’s disease or related dementias in different stages of the disease took part in this study. The selection of participants was made by the propositive sampling of maximum variation [36] to ensure the greatest possible variety of information according to age, gender, kinship, environment, paid external employment, external support, residence and care recipient’s stage of dementia (Table 1). The sampling considered the information on the characteristics of caregivers and families of patients with dementia provided by the censuses of the Portal for the Care of the Elderly Disabled and the censuses of people included in the Andalusian Regional Government’s Health Information System (Diraya) belonging to the Integrated Care Process: Dementia. In addition, the indications provided by health professionals about possible key participants in the study were considered. The participation of male caregivers in the focus groups was sought, although there were difficulties in identifying them because of their scarcity in the Mediterranean cultural context [37].

### 2.3. Data Collection

The focus group technique was used as a method of collecting information, which allowed for collective interaction, as well as the capture of some individual perspectives, and in essence facilitated the exploration of the topic of study. In order to avoid situations of discomfort or conflict, a suitable atmosphere was created during the development of the focus groups. In addition, the importance of confidentiality and respect among participants was emphasized. The groups were formed according to two basic variables that influence the process of caring for a person with dementia: the caregiver’s age and the level of dependency of the care recipient measured by the Barthel index [38], a scale that measures the level of autonomy of people in ten activities of daily living (Table 2). The nurses who collaborated in the study pre-screened the selected caregivers and their family members with dementia.

Two researchers (Sara Moreno-Cámara and Pedro Ángel Palomino-Moral), who were unknown to the participants, moderated the seven focus groups in different health centers in the province of Jaén, covering both rural and urban areas. These groups took place between July 2012 and February 2013. To populate the study, family nurses or case management nurses were contacted and offered voluntary participation in the study to the caregivers, and they agreed to collaborate in all cases. We adopted a conversational style of interview based on an interview script that was agreed upon in an expert meeting and included issues related to difficulties in care and the needs that would provide a response to these problems: rest, personal health, family support, feelings of self-efficacy/self-perception, time management, expectations, desires, social life, formal support, economy and residence. It is worth mentioning that the group of experts consisted of four people who work daily with caregiving families and research into this topic; the method of decision was by consensus. The interviews carried out in the different focus groups were recorded in audio; the average duration of the focus groups was 60 min.

### 2.4. Ethical Considerations

The study was approved by the Research Ethics Committee of Jaén and the Ethics Committee of the University of Jaén with the ethical approval number 2302201201. All standard ethical and data storage processes were adopted. An information document advertising the project was given to the caregivers who met the inclusion criteria. A signed consent form for each participant was obtained prior to conducting each focus group. To protect the confidentiality of participants, each transcript was assigned a code and all identifying information was deleted.

### 2.5. Data Analysis

The transcripts of the interviews were analyzed systematically using a thematic analysis approach, considering the semantic and pragmatic content and field notes [39]. Data analysis was conducted using Nvivo 8 software (QSR International, Victoria, Australia). Familiarization with the overall content was achieved through reading and rereading each transcript. During this time, notes were made about potential codes. The second phase of analysis involved the development of a list of codes that identified any feature of the data that was interesting and noteworthy. An inductive approach was adopted whereby coding was strongly linked to the data. The analysis continued as these themes were defined and redefined ensuring all data were represented. Analysis continued until all codes were found to form a coherent pattern within each theme. The themes were then clearly defined in terms of what they represented.

### 2.6. Validity and Reliability/Rigour

To enhance the credibility of the study, as well as the transferability of the results, rigorous data collection was carried out and the analysis followed, and textual references and their conceptualization were detailed. To establish another element of rigour and reduce the likelihood of introducing bias at the analysis stage [40], two researchers (Sara Moreno-Cámara and Lourdes Moral-Fernández) independently coded the transcripts and a third researcher (Antonio Frías-Osuna) was involved where there were discrepancies. The codes were then examined by all three researchers for ways in which they could be grouped to form themes or categories. In the final phase of analysis, another researcher (Laura Parra-Anguita) along with the previous ones reviewed the data and agreed upon extracts that were representative examples of the themes that they had identified.

## 3. Results

Two central categories of data analysis were identified: “Family caregivers’ needs related to the management of care for their family member with dementia” and “Personal needs of family caregivers of elderly people with dementia”. Each central category is composed of several sub-themes that will be detailed below.

### 3.1. Family Caregivers’ Needs Related to the Care of the Relative

#### 3.1.1. Instrumental Support

**Family instrumental support:** Caregivers feel they should have family instrumental support when they need it; “having someone else” who they can “trust fully” because they know they will react as she or he would. They need someone to help them with more physically demanding care, especially for older and/or sick caregivers.

“Knowing that a person is going to react the same way you do.” (Martos)

“Well, here most of them are old people, and others who are not so old, with a disability of 55%, and then you have your disability, but at the same time you have to take care of a person who is 84 years old. We need someone to help us.” (San Felipe)

On the other hand, the caregivers need someone to replace them at certain moments, even though they sometimes feel bad about asking for help. Caregivers say that the care of a family member with dementia “takes another form” if the care is shared with the rest of the family.

“My daughter is 14 years old, and I ask her to stay with Grandma, but she starts to complain; what am I to do?” (Torres)

“At first, I took care of my mother on my own, but since it got worse, I spoke to my brothers and thanks to them we are doing very well.” (Martos)

#### 3.1.2. Formal Support

**Instrumental support from paid and non-professional caregivers:** Family caregivers also value aid from paid external instrumental support. These external caregivers help in tasks of caring and household chores.

“I have the advantage of the girl who goes for an hour in the morning and later, for another hour, in the evening to put him to bed.” (Arjona)

**Respite services:** Family caregivers report that they need respite services; i.e., a place where they can leave their family member with dementia for a limited period, either at a regular time or periodically. They recognize the benefits they would gain from using these services, but they limit their use because of their beliefs, social pressure and respect for the care recipient’s preferences.

“I would love to have a place in which, for example, I have to go, I don’t know, that I have a meeting, or I want to have a coffee with my friends, that means I’m going to leave him there comfortably for 2 h and then pick him up later.” (Martos)

“If I took him to a nursing home for a month, it’d do me a lot, although I don’t know. I wouldn’t feel comfortable.” (Torres)

**Support from socio-sanitary professionals:** Family caregivers need the support of reference socio-sanitary professionals and demand regular follow-ups from the sick person. They highlight the importance of professionals responding quickly and effectively at critical moments, and of having the same professional care for their family member with dementia. In addition, they underline the humane and patient treatment they should have. They emphasize the need for professionals to learn more about dementia and can inform and teach them.

“They (socio-sanitary professionals) should go at least once a month; it’s not that they don’t show up, they don’t show up for anything.” (Andújar)

“I am thrilled with this. Because when it has really been necessary, they throw themselves into it immediately.” (Andújar)

**Support from social and health organizations:** Caregivers need the support of social and health organizations to speed up the process of diagnosis of their family member and exhaustive and professional assessments from social and health personnel to intervene and provide effective treatments to their family member with dementia.

“When they admit you into hospital, there are nurses who are charming, but there are others who do not have much endurance.” (La Magdalena)

“It takes a long time while they tell you what the disease is, among doctors, with this and that, and then they diagnose it to her.” (La Magdalena)

**Access to resources:** Caregivers stress the importance of public services being better resourced to facilitate infrastructural adaptations, physical support from external caregivers and the provision of technical assistance to those in need, and of resources arriving at the right time.

“I know that the resources they have are very limited, but I, for instance, asked for things and as they didn’t have them, I felt very overwhelmed.” (Martos)

#### 3.1.3. Training

**Practical training in care oriented towards meeting the basic needs of the care recipient:** Caregivers report that they need general information about dementia and the resources available, especially at the beginning of care and throughout the care process, due to the course of the disease and progressive changes in the care recipient. They stress that at the beginning of care they do not know where to turn and feel lost.

“Explain to us well how to do things to make them easier for us.” (La Magdalena)

“Having basic information about those daily medications; I think this is very important together with what that women said about holding the patient. It has done a lot for me.” (Andújar)

“(Training) Now and at the beginning, because it varies very much, the change that has taken place is incredible.” (Martos)

“They said to me: “this way” and the person gets up more easily, for them and for you.” (Andújar)

**Training to manage special care situations and behavioural problems:** Family caregivers also consider the importance of training in specific care when people with dementia are receiving specific treatment. Specifically, they ask for training to manage their family member’s behavioral problems.

“I didn’t know where to pick that (urinary catheter) up, so I tried to connect it; I didn’t know how to do that!” (Andújar)

“To know how to react before a specific situation or, say, if she goes on and on with this so many times, how am I to react?” (Torres)

### 3.2. Personal Needs of Family Caregivers of Elderly People with Dementia

#### 3.2.1. Own/Personal Life

**Disconnect from care:** Caregivers feel “tied down”, like slaves of the care recipient, and miss the freedom they once had and have lost by providing care. They point out that they need time for themselves: to rest, to get out and disconnect from care. They see the little time they can devote to “covering other spaces, such as marriage or children” as a theft from the care of their family member and that “the space that is always hollow is the staff”. As a result, caregivers become careless and stress-laden.

“There comes a time when you have no life because you’re looking out for them all day.” (La Magdalena)

“But if you invest that time, and it is true that it is for you, I, in my case, I don’t disconnect, although if I leave, although I go out, my mind is where it shouldn’t be, and it is with her (her mother), no, not me.” (Martos)

**Free time, leisure and social life:** Due to their dedication to care, caregivers have given up the things they would like to do and even neglected carrying out daily tasks. They need to take the time for themselves and do what they used to do.

“Yes, I miss going out, running errands, going out unhurriedly.” (San Felipe)

“I need that time for myself, for me, as a person to be with my husband, or to enjoy being with friends.” (Martos)

#### 3.2.2. Emotional Support

Caregivers say they need emotional support; others, especially family members, show appreciation, understanding and concern.

“At least get them to understand you, try and put them into your position.” (Andújar)

#### 3.2.3. Acknowledgment by Others

Likewise, caregivers also need recognition of their caregiving labor and sacrifice, including by the care recipient.

“I’ve sacrificed myself and my family, and, nobody gives you the merit.” (Arjona)

“And now she looks at me like dirt, nothing, she looks at me worse than at a maid (her mother), and cries.” (San Felipe)

#### 3.2.4. Satisfaction with Care Labor

Caregivers need to feel that they are doing the right thing to achieve well-being and satisfaction. They try to act according to their moral values to “have a clear conscience”.

“When they are well is when we are well; when they are well, we’re happy.” (Martos)

“I know what I’m doing to my mother is well done: that means I have a clear conscience.” (El Valle)

#### 3.2.5. Psychological Adjustment

**Effective coping with the disease:** Caregivers report that they need to cope with the illness adequately to be “psychologically prepared for what is to come and to endure”.

“We are unable to understand that they don’t do it to us, it’s just that they don’t know.” (Martos)

“That is what irritates me most: she spends all day screaming, day and night.” (Andújar)

**Management of stress and feelings derived from care labor:** The cognitive-behavioral problems of the caregiver’s relative (resistance to being cared for, aggressiveness, distrust, runaways, repetition of words, forgetfulness, etc.) are an important source of stress for caregivers. Therefore, they need to manage the stress and feelings of caring for their family member with dementia, such as guilt, insecurity and helplessness, among others, which create discomfort for themselves and their environment.

“Every day I put her to bed, I ask the Lord to take her away, every day, and when I got up I regretted it, thinking how bad I am; that discomfort, for me, has been the worst.” (Andújar)

“I sometimes feel grim, sad to see decadence and I am many times fearful when I forget something, frustrated, very impotent. I don’t know how many times I’ve felt that tension.” (Martos)

#### 3.2.6. Selfcare

**Health:** Family caregivers report that they need to maintain their health in order to provide appropriate care for a family member with dementia. Previous illnesses of the family caregiver and health problems resulting from the care make it difficult to care for the person with dementia and affect the caregiver’s well-being.

“I don’t do any exercise, I don’t go out, I only use the car, because of course, I go rushing, I go running, the fact is I don’t have time; therefore, I feel as if I’m choking, I need to breathe.” (Martos)

“I’ve been at it for 6 or 7 years and each day gets worse; I’m at a time that I need to be looked after, too.” (San Felipe)

“Physically, I have pains in the leg, waist, I’m continuously on alert and I don’t sleep well.” “I also have stomach trouble, which before I didn’t, and I have headaches, tension and stress, too.” (Martos)

**Rest:** The caregiver’s continuous state of alertness, constant concern and behavioral problems directly influence the caregiver’s rest and his or her performance as a caregiver.

“I have an obsession, I don’t sleep well at night, I have to take my medication.” (El Valle)

“I don’t sleep at night, and I feel whacked, I am collapsed, I am not useful for anything, I lack any strength.” (Torres)

## 4. Discussion

Our results reflect and expand on the evidence from previous studies on the wide range of needs of family caregivers of elderly people with dementia [41,42] and the interrelationship between these needs [43], focusing on the well-being of the caregiver—his or her own and that of his or her environment. Therefore, our results support the need to develop comprehensive interventions that include more than one need.

Interventions targeted at the confluence between the caregivers’ needs identified in the literature and those felt by caregivers appear to be more effective [7]. In this sense, our results support the prioritization of interventions with enough evidence of their effectiveness on the emotional health of caregivers [44] which coincides with the caregivers’ perceived needs. We suggest the importance of psychoeducational interventions [44] aimed at increasing the knowledge and skills of family caregivers in the care of the elderly people with dementia and in their self-care.

Given that, in Mediterranean culture, the care of a family member is considered an obligation and is mediated by social pressure [13,30,31], it is consistent that there is a discrepancy with the needs of caregivers in other contexts. As a matter of fact, some needs identified in other studies [45], such as family negotiation, time management, care planning, negotiating with the health care system and seeking resources outside the formal system, were not perceived by the caregivers of this research.

In our study, caregivers express their discomfort because of the lack of recognition of their work and the involvement of other family members in care, but do not express the need to acquire negotiation skills with the family to legitimize their role and get help. The family environment underestimates the work of the caregiver, possibly by normalizing their role, especially if they are women [46,47]. This issue goes unnoticed in studies conducted in other cultural contexts [26]. This suggests the importance of considering the family as a whole in interventions for caregivers of older people with dementia, as recommended by Losada et al. [48], as well as training them in family negotiation skills. In addition, the lack of time expressed by caregivers suggests the importance of training interventions in care planning and time management skills. Similarly, complaints by caregivers about some of the shortcomings of the formal system could be addressed through training in negotiating with the health care system.

In our study, caregivers do not identify possible sources of funding outside the formal system or the family. These external sources of resources, which include peer groups and new technology-based interventions, are important for information and emotional support [49]. In this sense, new technologies provide the advantage of being more compatible with caregiving in that little time is left to leave home and interact, and there is moderate evidence of their effectiveness in improving the social support and emotional health of caregivers [50]. The fact that these sources are not identified makes proactive interventions necessary to foster skills in the search for and use of these resources. In addition, it is also necessary to point out that our results highlight the importance of the effective management of existing social and health resources by health policies tailored to the needs of the population.

Likewise, our results largely coincide with those of a review about the needs of family caregivers in relation to information and training needs [26]. Specifically, there is coincidence with the sub-themes: “Practical training in care oriented towards meeting the basic needs of the care recipient” and “Training to manage special care situations and behavioural problems”. The importance of maintaining education and training throughout the entire care process is highlighted because of the progressive evolution of dementia. Therefore, interventions adapted to each illness stage of the person with dementia and the phase of the caregiving role could improve the caregivers’ quality of life [27,30].

### Limitations

The possible limitations of this study may be related to the background of the people participating in the study. The reality explored corresponds to a context where a caregiving model based on the high involvement of the family and scarce support of formal services prevail. The needs of caregivers in settings with other caregiving models may vary.

In addition, the collection of information through the focus group technique may have conditioned participants’ statements, as it is a method of collecting information that sometimes limits honest self-expression because of social acceptability.

## 5. Conclusions

This study provides the following conclusions: (1) the needs perceived by family caregivers, which are derived from the care of the dependent person and the self-care of the caregiver, are complex, changing and interrelated; (2) providing comprehensive interventions for the improvement of the caregiver’s emotional health encompassing more than one care need may be more effective; (3) providing psycho-educational interventions aimed at managing the diverse aspects of dementia and the care of caregivers themselves is important; (4) considering the family as a whole in interventions is valuable; and (5) there is a need for proactive interventions aimed at improving skills in negotiation, planning and the search for resources outside the family and the formal care system (where new technologies can be accommodated, allowing them to stay indoors).

Knowing the caregivers’ needs may enable the development of appropriate interventions to address these needs and reduce the negative consequences of family care for people with dementia.

## Figures and Tables

**Table 1:** Profile of the family caregivers participating in the study (*n* = 82).

**Gender**	
Female	91.50%
Male	8.50%
Average age (years)	58.33
**Kinship**	
Daughter	85.30%
Son	4.80%
Wife	7.30%
Husband	2.40%
**Environment**	
Rural	48.78%
Urban	51.22%
**Paid external employment**	18.30%
**Paid external instrumental support and use of respite services**	19.50%
**Residence in common with the caregiver**	70.70%
**Care recipient’s stage of dementia**	
Initial–intermediate (>60 Barthel Index, moderate dependency)	31.75%
Intermediate–advanced (<60 Barthel Index; severe dependency)	68.35%

**Table 2:** Distribution of the seven focus groups according to the age and sex of the family caregiver and the level of dependency of the care recipient.

Family Caregiver’s Age	Care Recipient’s Dependence	
	Barthel Index > 60 (Moderate Dependency)	Barthel Index < 60 (Total Dependency)
**<65 years old**	FG-3: HC El Valle (Jaén); 12 FC (1♂) FG-4: HC La Magdalena (Jaén); 5 FC	FG-1: HC Virgen de la Capilla (Andújar); 14 FC FG-5: HC Martos; 11 FC (1♂)
**>65 years old**	FG-6: HC San Felipe (Jaén) 12 FC (2♂)	FG-7: HC Torres; 15 FC (1♂) FG-2: HC Arjona; 13 FC (2♂)

FG: focus group, HC: health centre; FC: family caregiver; ♂: man.
